# *Mycobacterium haemophilum* and Lymphadenitis in Children

**DOI:** 10.3201/eid1101.040589

**Published:** 2005-01

**Authors:** Lesla E.S. Bruijnesteijn van Coppenraet, Edward J. Kuijper, Jerome A. Lindeboom, Jan M. Prins, Eric C. J. Claas

**Affiliations:** * Leiden University Medical Center, Leiden, the Netherlands; † Academic Medical Centre, Amsterdam, the Netherlands

**Keywords:** lymphadenitis, Mycobacterium haemophilum, real-time PCR, MGB, minor groove binder, research

## Abstract

Infections associated with *Mycobacterium haemophilum* are underdiagnosed because specific culture methods required for its recovery are not applied routinely. Using polymerase chain reaction (PCR) technology on fine needle aspirates and biopsied specimens from 89 children with cervicofacial lymphadenitis, we assessed the importance of *M. haemophilum*. Application of a *Mycobacterium* genus–specific real-time PCR in combination with amplicon sequencing and a *M. haemophilum–*specific PCR resulted in the recognition of *M. haemophilum* as the causative agent in 16 (18%) children with cervicofacial lymphadenitis. *Mycobacterium avium* was the most frequently found species (56%), and *M. haemophilum* was the second most commonly recognized pathogen. Real-time PCR results were superior to culture because only 9 (56%) of the 16 diagnosed *M. haemophilum* infections were positive by culture.

## Abstract 2

*Mycobacterium haemophilum* is the second most common pathogen in children with mycobacterial lymphadenitis.

Cervicofacial lymphadenitis is the most frequently encountered manifestation of nontuberculous mycobacterial (NTM) disease in children. In previous studies, *Mycobacterium avium* has been identified as the cause in >80% of the patients (*1*). Other mycobacterial species isolated from patients with lymphadenitis are *M. tuberculosis*, *M. malmoense*, *M. kansasii*, *M. scrofulaceum*, *M. intracellulare,* and *M. xenopi*. *M. haemophilum* has been described as the causative agent of lymphadenitis as well (*2*–*7*).

In an ongoing multicenter study in the Netherlands, the optimal treatment for NTM lymphadenitis is investigated. Diagnosis of mycobacterial infection is performed by using mycobacterial differential skin tests and fine needle aspiration biopsy. Biopsied specimens are subjected to acid-fast staining, mycobacterial culturing, and *Mycobacterium* genus–specific real-time PCR. Of 89 patients included in the study so far, mycobacterial species were identified in 55 cases, of which *M. avium* had been found in 50 patients (*8*). In addition, a mycobacterial infection without further identification was detected in 16 patients. An atypical mycobacterial infection was diagnosed in these patients because either acid-fast staining results were positive or the *Mycobacterium* genus–specific real-time polymerase chain reaction (PCR) was positive. Cultures or species-specific real-time PCR for *M. avium* and *M. tuberculosis* remained negative. Previously, an attempt to characterize these mycobacteria by sequence analysis of the genus-specific PCR fragment was successful in only 2 cases and showed *M. haemophilum* (*8*). In the current study, we further analyzed these uncharacterized mycobacteria.

*M. haemophilum* requires special growth conditions (*9*), and most of the diagnostic laboratories do not use these culture conditions. Furthermore, no molecular test is available to detect *M. haemophilum* directly in clinical materials. Therefore, *M. haemophilum* infection could be seriously underdiagnosed (*4*,*10*–*12*). In this study, we developed a species-specific real-time PCR to detect *M. haemophilum* directly in patient materials. This assay can show the actual prevalence of *M. haemophilum* in patients with mycobacterial lymphadenitis, but it could also be applied in other diseases and help elucidate the incidence and distribution of this species.

## Materials and Methods

### Bacterial Strains

Five *M. haemophilum* reference strains (all clinical isolates) were available for 16S and internal transcribed spacer (ITS) sequencing. Three strains were provided by the National Institute for Public Health and the Environment and 2 were provided by the Institute for Tropical Medicine (Antwerp, Belgium). The 25 mycobacterial strains used for specificity testing included *M. tuberculosis* complex, *M. kansasii*, *M. xenopi*, *M. avium*, *M. intracellulare*, *M. gordonae*, *M. chelonae*, *M. fortuitum*, *M. marinum*, *M. scrofulaceum*, and *M. malmoense.* A complete list of all strains (species and subspecies) has been published in Bruijnesteijn et al. (*8*). The strains were cultured in liquid Dubos medium at 35°C. The *M. haemophilum* strains were cultured at 30°C on solid Löwenstein-Jensen (LJ) medium with added iron citrate or in liquid Mycobacteria Growth Index Tube (MGIT) medium with X-factor-strip added (Becton-Dickinson, Alphen a/d Rijn, the Netherlands).

### Patients and Samples

#### Clinical materials were obtained from patients included in the CHIMED-study. In CHIMED (a multicenter nationwide study on real-time PCR as a diagnostic tool for atypical mycobacterial infections in children with lymphadenitis), treatment is randomized between surgical and medical treatment. Pediatric patients were included on the basis of clinical appearance of atypical mycobacterial lymphadenitis and a positive skin test (*13*,*14*). Fine needle aspirates were taken from affected lymph nodes. In patients who underwent surgical treatment, the removed lymph nodes were also submitted for investigation. A control group to assess the specificity of the assay was assembled from 50 patients with lymphadenitis caused by *Bartonella henselae*.

### Mycobacterial Diagnostics

Clinical materials were decontaminated with a Nalc-NaOH decontamination protocol (*15*). Auramine staining was performed on the decontaminated materials for detection of acid-fast rods. Standard mycobacterial culturing was performed at 35°C in liquid MGIT medium and on solid LJ medium. *M. haemophilum*–specific culturing was performed at 30°C on LJ medium with added iron citrate and in MGIT medium with X-factor-strip added. Mycobacterial species were identified by using the Inno-Lipa and more recently using the Inno-Lipa V2 assay (InnoGenetics, Gent, Belgium) (*16*). When no growth was detected after 12 weeks of incubation, the culture results were listed as negative. Samples were also investigated for the presence of other bacterial pathogens by conventional bacterial cultures and by PCR for *B. henselae* (*17*).

### Nucleic Acid Isolation

All clinical materials were processed as described in Bruijnesteijn et al. (*8*). DNA was extracted from bacterial strains and clinical materials according to the method of Boom et al. (*18*) with an overnight incubation with proteinase K.

### Primers and Probes

Genus-specific primers for sequencing the total ITS region of mycobacteria were described by Frothingham et al. (*19*). Primers described previously for a genus specific real-time PCR (*8*) were also used for sequencing a part of the ITS region directly from clinical materials. Using these primer sets combined, we applied a seminested PCR approach to increase the amount of amplicon. The part of the ITS region used in this real-time PCR shows considerable variation between mycobacterial species (*20*) (Figure). The primers used in the real-time PCR are genus-specific, and for the design of the *M. haemophilum–*specific minor groove binding (MGB) probe, the intraspecies and interspecies variation in the amplified ITS region was investigated. Alignments were made of the sequences of the *M. haemophilum* strains and of different mycobacterial species. The *Mycobacterium* genus–specific probe is described in Bruijnesteijn et al. (*8*).

**Figure:**
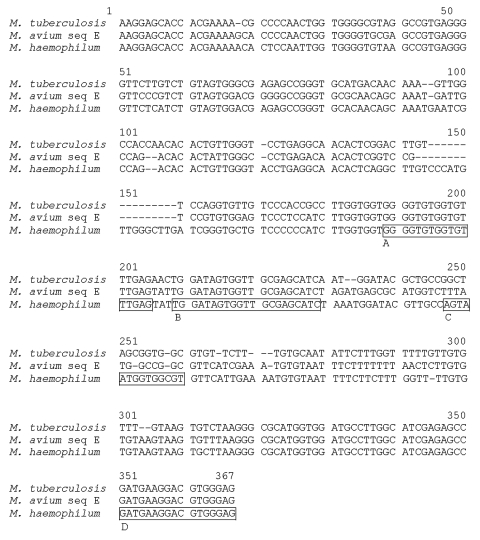
Alignment of internal transcribed spacers (ITS) and partial 23S sequences with primers and probes used for real-time polymerase chain (PCR) reaction. (nucleotides [nt] 1 to 301 make up the total ITS region; nt 302 to 367 are coding for partial 23S gene). The *Mycobacterium haemophilum* sequence was derived from 3 different patients, but no variation was found. A, forward primer for real-time PCR; B, *Mycobacterium* genus–specific probe; C, *M. haemophilum*–specific probe; D, reverse primer for real time–polymerase chain reaction.

The *M. haemophilum*–specific probe sequence was checked by using the primer 3 program (http://www-genome.wi.mit.edu/cgi-bin/primer/primer3_www.cgi/) (*21*) and oligo-analyzer 3.0 (http://biotools.idtdna.com/analyzer/) (IDT Biotools, Coralville, IA), to ensure minimal self-complementary and to prevent the presence of secondary structures. By using the unique features of the MGB group (*22*), a short and highly specific probe could be designed. The probe was designed on the antisense strand to ensure an A/T rich MGB-site. An NCBI BLAST search was performed to check the specificity of the probe. The primers were prepared by Biolegio (Biolegio, Malden, the Netherlands), and the MGB probe was generated by ABI (Applied Biosystems Inc, Nieuwekerk a/d IJssel, The Netherlands). The broad range primers P1 and P4 were used for 16S sequencing. Primers and probes are listed in Table 1, and their positioning in the genome is illustrated in the Figure.

**Table 1:** Sequences of oligonucleotides used in this study

Primer/probe sequence (5′–3′) target sequence		
ITS forward primer real-time PCR	GGGGTGTGGTGTTTGAG	Partial ITS
ITS reverse primer real-time PCR	CTCCCACGTCCTTCATC	Partial ITS
Forward primer Ec16S.1390p*	TTGTACACACCGCCCGTCA	Total ITS
Reverse primer Mb23S.44n*	TCTCGATGCCAAGGCATCCACC	Total ITS
16S forward primer P1†	TAACACATGCAAGTCGAACG	16S
16S reverse primer P4†	TCGTTGCGGGACTTAACCCAAC	16S
*Mycobacterium* genus–specific TaqMan probe	Fam-GGATAGTGGTTGCGAGCATC-Tamra	ITS
*M. haemophilum*–specific MGB-probe	VIC–ACGCCACCATTACT-MGB	ITS

^*^Primers published in (19). †Primers published in (*23*).

### Sensitivity Testing

A plasmid with the ITS equence of *M. haemophilum* was prepared by cloning the PCR product in a vector and was subsequently quantified (IQ corporation, Groningen, the Netherlands). Dilution series of this plasmid were tested in duplicate in the genus-specific and the *M. haemophilum*–specific real-time PCR.

### Sequence Analysis

After amplification, PCR products were subjected to a cycle sequencing reaction with the Big Dye Terminator Cycle Sequencing ready reaction kit (Applied Biosystems). Samples underwent electrophoresis and sequences were detected and analyzed on ABI model 310 DNA sequencer (Applied Biosystems).

### Real-time PCR

Real-time PCR was performed in 50 μL of reaction mixture consisting of 25 μL of 2x IQ supermix (Bio-Rad, Veenendaal, the Netherlands), 20 pmol of each primer, 12.5 pmol of the genus-specific probe or 10 pmol of the *M. haemophilum*–specific probe, and 10 μL template. The PCR thermal profile consisted of an initial incubation of 3 min at 95°C for activation of the enzyme, followed by 50 cycles of 30 s at 95°C, 40 s at 55°C, and 30 s at 72°C. Amplification, detection, and data analysis were performed with an iCycler IQ real-time detection system (Bio-Rad). The reaction mix and PCR profile were similar for both the genus-specific probe and the *M. haemophilum* probe.

Each DNA extract was tested by real-time PCR for the detection of the genus *Mycobacterium* and species *M. haemophilum*. As positive control for the genus-specific real-time PCR and extraction protocol, a dilution of *M. bovis* was used.

## Results

### Identification of *M. haemophilum* in Patient Material

In 16 (18%) of 89 patients from the CHIMED study, a mycobacterial infection was suspected, but initially no species identification could be established. After a positive signal was generated by the genus-specific real-time PCR and negative results from the cultures, the amplicons generated in real-time PCR were sequenced to determine the species. Sequencing of the ITS fragment formed in the real-time PCR was difficult, owing to the small amount of amplicon, but eventually the sequences of 4 patient samples were successfully derived. On 4 more samples, a seminested PCR was performed to increase the amount of specific amplicon. This enhancement of PCR resulted in the successful amplification and sequencing of all fragments. No variation was encountered between the ITS sequences of all 8 strains analyzed here. Because no *M. haemophilum* ITS sequences were available in the public databases, 3 complete ITS sequences from *M. haemophilum* strains isolated from different CHIMED patients were determined and submitted to the NCBI database (accession numbers AY579398, AY579399, and AY579400). After specific culturing, the identity of the strains was confirmed by comparing partial 16S-gene sequences to the sequences in the NCBI (http://www.ncbi.nlm.nih.gov/) and the RIDOM database (http://www.ridom.com/). A variable part of the 16S gene of 330 base pairs was analyzed, and a 100% agreement was obtained with 16S sequences of 7 available *M. haemophilum* strains, including ATCC 29548. The *M. haemophilum* strains had at least 4 mismatches in the analyzed 16S PCR fragment in comparison to other mycobacterial species; therefore, all these strains were *M. haemophilum*. The identity was also confirmed because of a minimum of 4 mismatches in the 16S fragment between the *M. haemophilum* sequence and other mycobacterial species.

### Application of Real-time PCR to the Recognition of *M. haemophilum*

The real-time PCR was designed to the ITS region. The same conserved primers were used as described previously. The obtained ITS sequences were used to select an *M. haemophilum–*specific probe.

The detection limit of the *M. haemophilum*–specific real-time PCR was assessed at 1 copy per reaction by using a dilution series of the plasmid standard. The mycobacterial genus–specific PCR was tested simultaneously with the *M. haemophilum*–specific PCR and resulted in the same analytical sensitivity. As determined previously, the sensitivity of the primer set in clinical materials was estimated to be 1,100 CFU in pus (*8*). Specificity testing of the *M. haemophilum–*specific real-time PCR with 25 other species and subspecies showed no aspecific reactions. All 50 *Bartonella*-positive samples from the control group remained negative in the real-time PCR assay as well.

Of 16 patients with evidence for *M. haemophilum* infection, 9 (56%) were positive on auramine staining, and 9 (56%) were positive in *M. haemophilum*–specific cultures. In addition, in 1 patient (6%), the pathogen was able to grow on and in normal mycobacterial cultures. Thirteen patients (81%) had positive specimens in mycobacterial genus–specific real-time PCR, 11 of which were also positive in the *M. haemophilum*–specific real-time PCR (Table 2). In contrast, 2 genus-specific *M. haemophilum–*negative specimens were positive in the *M. haemophilum*–specific real-time PCR. Thus, the 2 PCRs combined yielded 15 positive (94%) patients. These 4 samples with inconsistent results all had high threshold cycle values, indicating that the amount of bacterial DNA present was close to the detection limit of the assays. This finding was confirmed by retesting the samples 5 times. As expected, this resulted in 2 or 3 positive reactions for any sample that had undergone both assays. No correlation was found between the threshold cycle values in the real-time PCR assay and the culture or auramine-staining results. All 9 patients with positive specimens by auramine staining also had positive results in the real-time PCR assay. Three patients’ conditions were diagnosed by real-time PCR only. Only 1 patient had a positive culture while results of the real-time PCR or auramine staining remained negative. Real-time PCR on the isolate cultured from this patient resulted in a positive signal.

**Table 2:** Results of diagnostics tests of 16 *Mycobacterium haemophilum–*positive patients

*M. haemophilum–* positive patient	Acid-fast staining	Culture 30°C*	Genus-specific real-time PCR	*M. haemophilum–*specific real-time PCR
1	+	–	+	+
2	–	+	–	–
3	+	–	+	+
4	–	–	+	+
5	–	–	+	+
6	+	+	+	+
7	+	–	+	+
8	+	+†	+	+
9	+	+	+‡	–‡
10	+	+	–‡	+‡
11	+	–	+‡	–‡
12	–	+	–‡	+‡
13	–	+	+	+
14	–	–	+	+
15	–	+	+	+
16	+	+	+	+
Total positive patients	9	9	13	13

*Löwenstein-Jensen (LJ) medium with added iron citrate or liquid MGIT medium with X-factor-strip added. Cultures at 30°C were performed after storage for patients 1 to 6. †Patient material was also culture positive at 35°C. ‡Because of discrepant polymerase chain reaction (PCR) results with high threshold cycle values, the PCR was performed 5 times on these samples, which resulted in at least 2 specific positive signals for both PCRs on every sample. Therefore, the amount of mycobacterial DNA is estimated at the detection limit of the assay. The first obtained PCR result is described in the table.

The *M. haemophilum*–specific culturing method was less sensitive than the real-time PCR assay. The materials from the first 6 patients were cultured specifically for *M. haemophilum* after negative results were obtained from conventional culturing methods. The stored decontaminated materials were thawed and incubated at 30°C on enriched media. From these 6 patients, 2 samples (33%) yielded positive cultures. The materials from the 10 other patients were cultured directly and yielded positive results from 7 (70%) patients. *M. haemophilum*–specific real-time PCR was performed additionally on all positive cultures to confirm the specific growth of *M. haemophilum*.

From the 16 patients positive for *M. haemophilum*, 22 samples were collected: 9 tissue biopsy specimens and 13 fine needle aspirates. Of these samples, 19 (86%) were positive in the real-time PCR assay, while 16 (73%) samples yielded positive results in auramine staining with 11 positive (50%) or 9 positive (36%) results, respectively. No discrepancies were encountered in the real-time PCR assay when all samples instead of patients were considered. Application of the real-time PCR assay increased the diagnostic yield by 23%.

## Discussion

*M. haemophilum* was found to be the causative agent of lymphadenitis in 16 (18%) of the children included in this study. Despite the use of specific enriched culture mediums, only 9 (56%) of the 16 *M. haemophilum* infections were culture-positive. In contrast, the real-time PCR assay was positive in 15 (94%) patients.

*M. haemophilum* infection is not diagnosed frequently and is therefore not considered a common cause of lymphadenitis. However, most studies on children with mycobacterial lymphadenitis have not used optimized cultures for *M. haemophilum*, and infection with this species is therefore likely underdiagnosed. Nevertheless, differences in geographic distribution may also contribute to the variable prevalence of *M. haemophilum*. For instance, no *M. haemophilum* was found in children with atypical mycobacterial lymphadenitis in a study in Ohio (*24*), whereas in a study in Israel, *M. haemophilum* was found in 12 of 29 patients (*5*). Both studies used appropriate culture conditions for *M. haemophilum*. Another reason for an underestimation of the occurrence of *M. haemophilum* infections is the misleading positive skin test. *M. haemophilum* can induce similar reactions in the Mantoux test as *M. tuberculosis* and could be misdiagnosed when no positive cultures are obtained (*4*,*5*).

The natural source of *M. haemophilum* infection is unknown. Its geographic distribution appears to be related to the occurrence of large bodies of water (*6*). A few natural reservoirs have been suggested (*25*–*27*), but studies focusing on the environmental reservoirs of NTM tend to culture without optimized conditions for *M. haemophilum*, which may be the reason the organism is rarely found. The temperature for culture is often too high (*28*,*29*), cultures do not contain hemin or iron citrate, or the incubation time is too short (*30*). Only 1 study detected *M. haemophilum* in water distribution systems, although the culture method was not optimal (*26*). Therefore, *M. haemophilum* may be widely distributed and present in several natural reservoirs; water is the most likely one (*12*).

*M. haemophilum* is slowly growing, iron dependent, and has an optimal growth temperature from 30°C to 32°C. It is unable to grow on routine media such as Lowenstein-Jensen, Middlebrook 7H9 and 7H10, or BACTEC broth. Media used to recover *M. haemophilum* on primary isolation include commercially available solid media or broth enriched with ferric ammonium citrate or hemin (*31*). Chocolate agar and lysed blood agar are mentioned as inexpensive and suitable alternatives (*32*,*33*). Little is known about the sensitivities of direct culturing of clinical materials for the recovery of *M. haemophilum*, and not all media have equal capacity for stimulating the growth (*34*).

Because application of culture conditions specific for *M. haemophilum* are not likely to become standard in clinical microbiologic laboratories, including this specific diagnosis might be useful for molecular methods. A species-specific real-time PCR was developed to identify *M. haemophilum* directly in patient materials. Because *M. haemophilum* was not expected to be an important pathogen, no specific culturing was applied initially. After the recognition of *M. haemophilum* by molecular methods, the culture methods were optimized, which resulted in 70% positive cultures. Additionally, all stored decontaminated materials from culture-negative specimens were recultured under *M. haemophilum*–specific conditions. Most likely because of these additional freezing and thawing steps, cultures were less sensitive for these materials and resulted in 33% positive cultures.

Identification of *M. haemophilum* in patient materials was performed by 16S sequencing (of cultures) and ITS sequencing. Two versions of a commercial reverse line hybridization assay (the Inno-Lipa assay and the V2 Inno-Lipa assay) were also used for the recognition of *M. haemophilum*, but these tests can only be applied on cultured isolates. The V2 Inno-Lipa can identify *M. haemophilum* by a specific probe, which was absent in the previous version of the Inno-Lipa assay. The reactions were uniformly positive only for *M. haemophilum* in the V2 Inno-Lipa.

The design of the real-time PCR MGB probe was based on the ITS sequences that were obtained from the patient materials and reference strains. An MGB probe enables specific detection of the target by using a shorter sequence than that of a TaqMan probe or a molecular beacon.

Sequencing of the ITS amplicons from the genus-specific real-time PCR on patient samples was difficult because of the small amounts of target sequence. To enhance the specific amplification, a seminested PCR was applied. Of the 8 clinical samples from which sequences were obtained, 4 samples also yielded positive cultures once the culture protocol was optimized. The ITS and 16S sequences derived from the cultured isolates confirmed the authenticity of the identified pathogen.

In this study, both fine needle aspiration and excisional biopsy were not applied as treatment options but as diagnostic procedures. Complete surgical excision of the affected lymph nodes is considered as the treatment of choice for atypical mycobacterial lymphadenitis (*1*,*35*,*36*). However, surgical excision leaves scarring and carries the risk of damaging branches of the peripheral facial nerves (*37*,*38*). Antimicrobial therapy as a conservative treatment is currently the topic of our study. Incision and drainage increase the risk for sinus tract formation or recurrence of infection (*33*,*35*). This risk also applies to fine needle aspiration, but the usage of fine needle aspirate for PCR will provide a rapid diagnosis and thereby allow treatment to begin earlier and thus lower the risk for complications.

In conclusion, for detecting and identifying *M. haemophilum*, real-time PCR is a sensitive and specific assay suitable for direct application on clinical materials. In this study, by using the real-time PCR, *M. haemophilum* was shown to be an important pathogen involved in lymphadenitis. Because of special growth requirements, the clinical spectrum of diseases associated with *M. haemophilum* is largely unknown. Real-time PCR may be particularly useful for testing clinical samples such as sputum, cerebrospinal fluid, and synovial fluid for *M. haemophilum* to determine the role of *M. haemophilum* in more detail.
